# Outcomes of kidney transplant recipients admitted to the intensive care unit: a retrospective study of 200 patients

**DOI:** 10.1186/s12871-019-0800-0

**Published:** 2019-07-17

**Authors:** Damien Guinault, Arnaud Del Bello, Laurence Lavayssiere, Marie-Béatrice Nogier, Olivier Cointault, Nicolas Congy, Laure Esposito, Anne-Laure Hebral, Olivier Roques, Nassim Kamar, Stanislas Faguer

**Affiliations:** 1 Département de Néphrologie et Transplantation d’organes, Unité de Réanimation, Hôpital Rangueil, Centre Hospitalier Universitaire de Toulouse, 1, avenue Jean Poulhes, 31059 Toulouse, France; 2 Laboratoire d’Immunologie, Hôpital Rangueil, Centre Hospitalier Universitaire de Toulouse, F-31000 Toulouse, France; 3 Université Paul Sabatier, Toulouse III, F-31000 Toulouse, France; 4 Institut National de la Santé et de la Recherche Médicale, U1043, IFR–BMT, CHU Purpan, Toulouse, France; 5 Institut National de la Santé et de la Recherche Médicale, Institut des Maladies Métaboliques et Cardiovasculaires, U1048 (Renal Fibrosis lab), and French Intensive care Renal Network (F.I.R.N), Toulouse, France

**Keywords:** Renal transplantation, HLA immunization, Intensive care unit, Outcomes

## Abstract

**Background:**

Risk of over-immunosuppression or immunization may mitigate the overall and long-term renal outcomes of kidney transplant recipients (KTR) admitted to the ICU in the modern era but remain poorly described. Thus, there is an unmet need to better characterize the survival of KTR admitted to the ICU, but also the renal and immunological outcomes of survivors.

**Methods:**

Retrospective observational study that included 200 KTR admitted between 2010 and 2016 to the ICU of a teaching hospital (median age 61 years [IQR 50.7–68]; time from transplantation 41 months [IQR 5–119]). Survival curves were compared using the Log-rank test.

**Results:**

Mortality rates following admission to the ICU was low (26.5% at month-6), mainly related to early mortality (20% in-hospital), and predicted by the severity of the acute condition (SAPS2 score) but also by Epstein Barr Virus proliferation in the weeks preceding the admission to the ICU. Acute kidney injury (AKI) was highly prevalent (85.1%). Progression toward chronic kidney disease (CKD) was observed in 45.1% of survivors. 15.1% of survivors developed new anti-HLA antibodies (donor-specific antibodies 9.2% of cases) that may impact the long-term renal transplantation function.

**Conclusions:**

Notwithstanding the potential biases related to the retrospective and monocentric nature of this study, our findings obtained in a large cohort of KTR suggest that survival of KTR admitted in ICU is good but in-ICU management of these patients may alter both survival and AKI to CKD transition, as well as HLA immunization. Further interventional studies, including systematic characterization of the Epstein Barr virus proliferation at the admission (i.e., a potential surrogate marker of an underlying immune paralysis and frailty) will need to address the optimal management of immunosuppressive regimen in ICU to improve survival but also renal and immunological outcomes.

## Background

About 10% of kidney transplant recipients (KTR) experience a life-threatening disease requiring admission in an intensive care unit (ICU) [1–7]*.* Most admissions occur more than 6 months after the renal transplantation [3–5, 8, 9]. The main causes of admission were acute respiratory failure (ARF) and septic shock, followed by cardiovascular complications, acute kidney injury (AKI), drug-related complications and neoplasia [4, 10].

In-hospital mortality after admission to the ICU is mostly related to the cause of admission and the number of organ failures at presentation, whereas the characteristics of the renal transplantation are not associated with the outcomes [3, 4, 6]. Whether these findings hold true in the modern era characterized by an increase of transplantations at high risk of surgical and immunological complications and with widespread use of prophylaxis for opportunistic infections remain to be addressed.

KTR are at higher risk of severe AKI in the ICU, compared to unselected critically ill patients [3, 11], and up to 40% of KTR will required renal replacement therapy (RRT). AKI is now recognized as a cause of chronic kidney disease (CKD), and estimated glomerular filtration rate (eGFR) before the injury is a strong predictive factor of progression toward CKD [12]. In old studies, the renal outcome was poor, ranging from 20 to 30% of patients with eGFR decline after ICU stay. KTR that develop AKI have a relative risk of graft loss of 3.2, and up to 20% of patients will ultimately lose their renal graft [13]*.*

Similarly to unselected critically ill patients, renal outcome of KTR results from the combination of the underlying CKD (*i.e*, basal eGFR), the use of nephrotoxicants in the ICU, and episodes of ischemic, hemodynamic or septic AKI [3, 4, 14]. In the setting of renal transplantation, immunological injuries may also promote the progression of graft dysfunction observed after ICU admission. Indeed, the withdrawal of immunosuppressive drugs and/or red blood cells (RBC) transfusion may lead to the development of de novo donor-specific antibodies during or after the stay in the ICU [15, 16]. This may reduce the graft survival in ICU survivors and reduce the access to a subsequent transplantation in patients who lost their graft function. To date, no study accurately assessed the immunological outcome in KTR admitted in ICU.

In this study, which included a large cohort of 200 KTR admitted in ICU over a 6 years period, we aimed to identify the predictive factors for in-hospital mortality, to characterize the predictive factors of progression from AKI of CKD, and to assess the risk of anti-HLA immunization, two factors associated with long-term survival.

## Patients and methods

In this retrospective single-center study, we included all KTR admitted between January 2010 and June 2016 to the ICU of the Department of Nephrology and Organ Transplantation at the University Hospital of Toulouse (France), a 10-bed tertiary care ICU backed by a 30 beds-unit of solid organ transplantation, and with 24-h-a-day intensivist.

To be included in this study, patients met the following criteria: (i) over 18 years of age, (ii) to have received a renal transplantation before the admission, and (iii) an admission to the ICU for acute conditions. Patients admitted for a close monitoring just after the renal transplantation, and those with known irreversible graft failure, were excluded from the analysis. Only the first admission was reported.

According to the French law related to retrospective observational studies and our Institutional Review Board (University Hospital of Toulouse – Office of Research, Development and Innovation), the need for written consent was waived.

The primary objective of the study was to identify the predictive factors of death in the hospital of KTR admitted to the ICU. Secondary objectives were the characterization of the risk to develop AKI to CKD transition and to acquire HLA immunization.

### Clinical characteristics

Clinical and biological data were collected from the computerized charts of the patients. The following parameters related to transplantation were collected: age at transplantation, cause of renal disease, immunosuppressive regimen, episodes of biopsy-proven antibody or T-cell-mediated rejection (ABMR and TCMR), EBV or CMV replication in blood or BK virus shedding in urine within the 6 months preceding the admission to the ICU. The parameters related to the ICU stay included age at admission, gravity scores, causes for admission, infections, organ failures, RBC transfusions, and immunosuppression management. Changes in immunosuppression were not standardized in our ICU and were left at the discretion of each physician. CKD was assessed at month 1 and 6 in survivors according to the CKD KDIGO staging [17].

### Immunological tests

Assessment of anti-HLA immunization was performed according to our institutional protocol (i.e. every 12 months or after events at risk of anti-HLA immunization like RBC transfusion, acute rejection), with the Luminex° technique. A baseline value of > 500 was considered positive.

### Statistical analyses

Continuous variables are reported as their median and interquartile ranges (IQR), and discontinuous variables as numbers and percentages. Univariate analyses of in-hospital mortality were performed using the Mann–Whitney or Fischer’ exact tests, as appropriate. Multivariate analyses were performed using a step-by-step logistic regression model. All variables associated with in-hospital survival by univariate analysis (*p* < 0.1) were included in the multivariate analysis. Survival curves were drawn according to the Kaplan-Meier method and compared with the Log-Rank test (univariate analysis). Statistical significance was assumed at *p* < 0.05. Statistical analyses were performed using the GraphPad Prism6 (San Diego, CA, USA) and Xlstat softwares (Addinsoft, Paris, France).

## Results

**Fig. 1:**
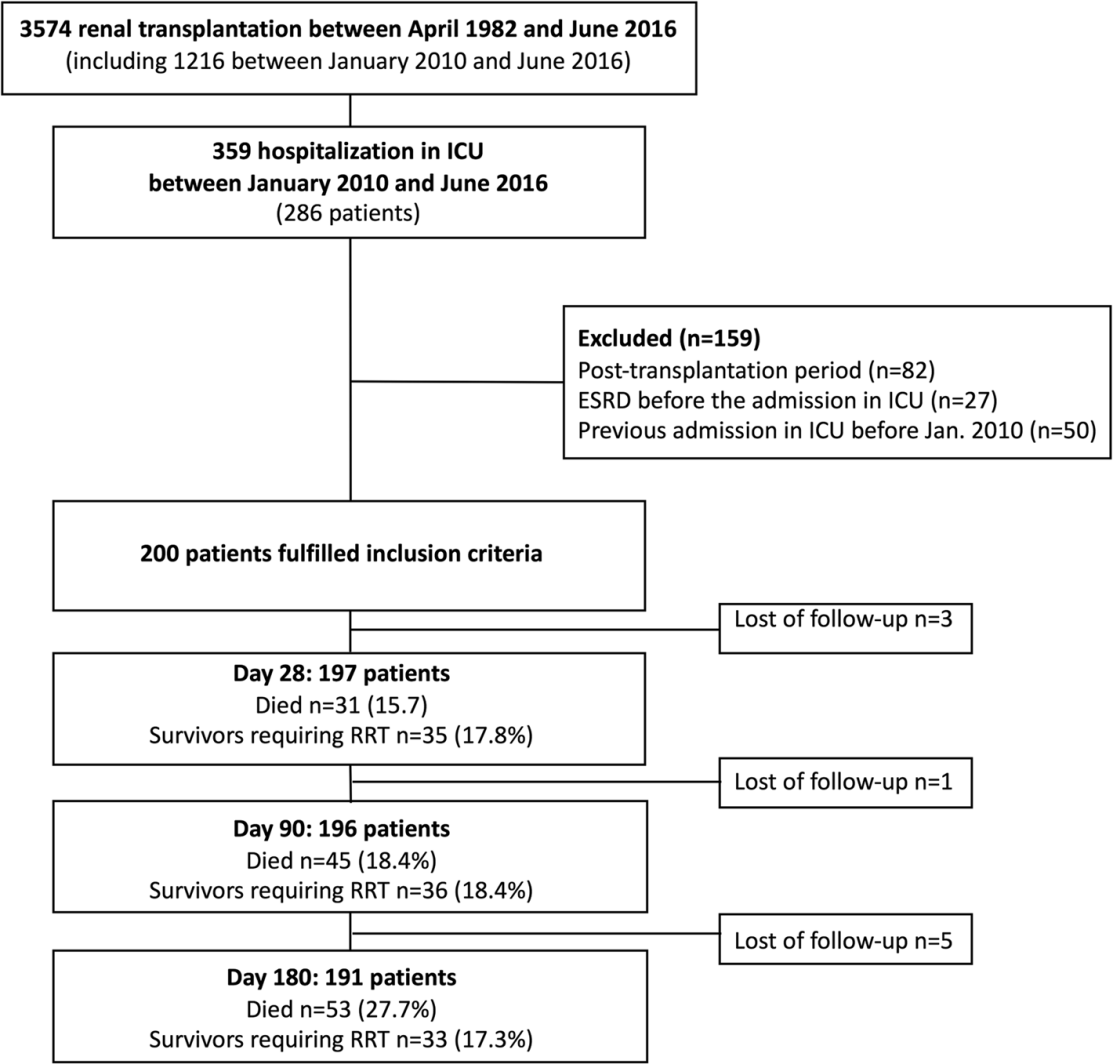
Flowchart of the study

From January 2010 to June 2016, 286 KTR were admitted at least once to our ICU, including 200 that met the inclusion criteria (median age 61 years [IQR 50.7–68]; male to female ratio 126/74; time from transplantation 41 months [IQR 5–119]; Fig. 1). During this period 1240 patients received renal transplantation in our transplantation unit, and 95 out of these 1240 (7.7%) were admitted to the ICU. Main characteristics of the transplantation and at the admission are summarized in Tables 1 and 2.

**Table 1:** Characteristics of the 200 kidney transplant recipients before admission in ICU

Characteristics	Total *N* = 200	Survivors *N* = 160	In-hospital death *N* = 40	*p*
Age (n, %)	61 [51–68]	60 [49–66]	65 [58–70]	0.07
Male gender (n, %)	126 (63)	97 (60.6)	29 (72.5)	0.20
Comorbidities (n, %)				
Diabetes mellitus	71 (35.5)	56 (35)	15 (37.5)	0.81
Heart disease (left ventricular systolic function < 45%)	97 (48.5)	73 (45.5)	24 (60)	0.11
Peripheral arterial disease	23 (11.5)	60 (37.5)	24 (60)	0.01
Solid cancer	11 (5.5)	6 (3.8)	5 (12.5)	0.05
Active hematological malignancy	9 (4.5)	6 (3.8)	3 (7.5)	1.00
Transplantation characteristics (n, %)				
Deceased donor	15 (7.5)	14 (8.7)	1 (2.5)	0.31
First kidney transplantation	166 (83)	136 (85)	30 (75)	0.20
Induction				
No	30 (15)	23 (14.4)	7 (17.5)	0.62
Polyclonal antibodies	90 (45)	74 (46.3)	16 (40)	0.58
IL2R blocking agents	72 (36)	57 (35.6)	15 (37.5)	0.85
Immunosuppressive regimen				
Steroids	184 (92)	148 (92.5)	36 (90)	1.00
CNI	161 (80.5)	131 (81.8)	30 (75)	0.49
Tacrolimus	123 (61.5)	104 (65)	19 (4.8)	0.97
Antimetabolites	163 (81.5)	137 (85.6)	26 (65)	0.008
MMF				
mTOR inhibitors	36 (18)	29 (18.1)	7 (17.5)	1.00
Belatacept	8 (4)	6 (3.8)	2 (5)	0.65
CNI, MPA, Steroids association	xx	xx	xx	
History of acute rejection				
Antibody mediated rejection	26 (13)	19 (11.8)	7 (17.5)	0.43
T-cell mediated rejection	34 (17)	28 (17.5)	6 (15)	0.82
Viral status before the admission ^#^ (n, %)				
Blood *Cytomegalovirus* detection (*n* = 142)	16 (11.3)	9/107 (8.4)	7/35 (20)	0.07
Blood *Epstein-Barr virus* detection (n = 127)	39 (30.7)	24/96 (25)	15/31 (48)	0.02
Urine BK virus shedding (*n* = 115)	28 (24.3)	18/90 (20)	10/25 (40)	0.04
Immunological status before the admission in ICU (n, %)				
Anti-HLA immunization (*n* = 188)	71 (38)	59/152 (38)	12/36 (33)	0.57
Donor-specific antibodies	23 (12.2)	19 (12.5)	4 (11.1)	1.00

Abbreviations: *IL-2R,* interleukin-2-receptor; *CNI*, calcineurin inhibitors; *MPA*, mycophenolic acid; *mTOR*, mammalian target of rapamycin; ICU, intensive care unit

**Table 2:** Characteristics of the 200 kidney-transplant recipients at the admission in ICU and during ICU stay

Characteristics	Total *N* = 200	Survivors *N* = 160	In-hospital death *N* = 40	*p*
Time since kidney transplantation (months)	40 [5–119]	37 [4–118]	54 [12–122]	0.14
Causes of admission (n, %)				
Acute respiratory failure	55 (27.5)	42 (26.3)	13 (32.5)	0.43
Septic shock	53 (26.5)	42 (26.3)	11 (27.5)	0.84
Unplanned surgery	46 (23)	40 (25)	6 (15)	0.21
Cardiogenic shock	18 (9)	12 (7.5)	6 (15)	0.21
Acute neurological condition	12 (6)	9 (5.6)	3 (7.5)	0.71
Acute kidney injury	10 (5)	10 (6.3)	0 (0)	0.22
Others	6 (3)	5 (3.1)	1 (2.5)	1.00
Sepsis at the admission (n, %)				
Overall	114 (57)	89 (55.6)	25 (63)	0.48
Lung	57 (50)	47 (52.9)	10 (40)	0.70
Urinary	26 (22.8)	23 (25.8)	3 (7.5)	0.30
Peritonitis	24 (21)	14 (15.7)	10 (25)	0.01
Others	7 (6.1)	5 (5.1)	2 (1.4)	0.64
Organ failures (n, %)				
SOFA score	6 [4–8]	6 [4–8]	8 [7–10]	< 0.001
SAPS2 score	50 [39–63]	48 [37–59]	67[57–77]	< 0.001
Acute kidney injury (KDIGO)				
stage 1	49 (24.5)	42 (26.3)	7 (17.5)	
stage 2	8 (4)	6 (3.8)	2 (5)	
stage 3	113 (56.5)	86 (53.8)	27 (67.5)	0.008
Renal replacement therapy	103 (51.5)	76 (47.5)	27 (67.5)	0.03
Mechanical ventilation	107 (53.5)	78 (48.8)	29 (72.5)	0.008
Vasopressive drugs	97 (48.5)	68 (42.5)	29 (72.5)	0.001
Normalized prothrombin time < 50%	11 (5.5)	5 (3.1)	6 (15)	0.01
RBC transfusion	142 (71)	109 (68.1)	33 (82.5)	0.08
Immunosuppressive regimen (n, %)				
No change	45 (22.5)	45 (28.1)	0 (0)	0.02
Increased dose of steroids	136 (68)	100 (62.5)	36 (90)	0.2
Withdrawal of CNI	40 (20)	25 (15.6)	15 (37.5)	0.004
Withdrawal of antimetabolites	61 (30)	48 (30)	13 (32.5)	0.85

### Transplantation characteristics

Immunosuppressive regimen included induction therapy in 162 patients. At admission, patients received a combination of calcineurin inhibitors (81.3%; tacrolimus 74%), antimetabolites (82.3%; mycophenolic acid 79.3%), mTOR inhibitors (19.2%) and/or steroids (92.9%). Overall, 123 patients received an immunosuppressive regimen including steroids, mycophenolic acid and calcineurins inhibitors.

Twenty-six (13%) and 34 (17%) out of the 200 patients had presented with an ABMR and TCMR before the admission in the ICU (12 (6%) developed both ABMR and TCMR). When available within the 6 months preceding the admission to the ICU, CMV and EBV replication in the blood were observed in 16/142 patients (11.3%), 39/127 (30.7%), respectively.

### Characteristics in the ICU

At admission, median SOFA gravity score was 6 [IQR 4–8]. Main causes of admission were ARF (27.5%), septic shock (26.5%), post-operative period (peritonitis, hemorrhage, 23%), acute neurological disorder (6%), AKI requiring RRT (5%) or cardiac arrest (1%).

Altogether, 114/200 admissions (57%) were related to an infection (lungs (28.5%), urine (13%), gut (12%) or other (3.5%)). Pyogenes-related infections were diagnosed in 101 patients, whereas aspergillosis, candidemia, pneumocystosis and CMV infections were identified in 8, 8, 7 and 5 patients, respectively. Multiple infections were identified in 29 patients (14.5%).

Median time of hospitalization in the ICU was 5 days [IQR 2–10]. Among the 200 individuals, 107 (53.5%) required mechanical ventilation, and 97 (48.5%) required vasopressive drugs. One hundred and seventy-one patients (85.5%) developed AKI, including 113 (56.5%) with KDIGO stage 3 AKI and 103 (51.5%) that required RRT. One hundred and forty-two out of the 200 patients (71%) benefitted from RBC transfusion.

### Management of immunosuppression in the ICU

At the admission, median residual concentration of tacrolimus and ciclosporine-A were 6.6 ng/mL [IQR 4.6–11.2] and 127 ng/mL [IQR 77–223], respectively. Prednisolone was switched to hydrocortisone (150 to 300 mg/day) in 42 patients, including 35 with septic shock. Calcineurin inhibitors were withdrawal in 40 patients (20%) and concentration targets were reduced in the others (tacrolimus residual 5–8 ng/mL; cyclosporine-A residual (70–220 ng/mL). Antimetabolites were stopped in 61/163 patients (37.4%), including 19 with sepsis. In 48 out of these 61 patients, the dose of steroids was increased. Overall, the immunosuppressive regimen was modified in 155 patients (77.5%).

### Predictive factors of in-hospital mortality

**Fig. 2:**
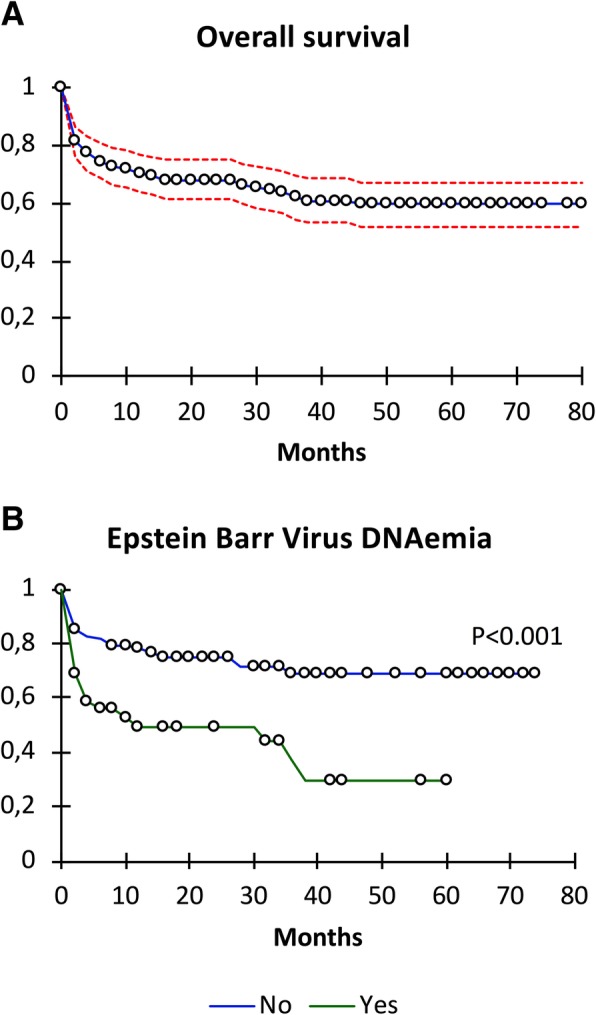
Survival curves following admission to the ICU. **a**. Overall population. **b**. Survival according to EBV replication before admission

Of the 200 patients, 25 (12.5%), 40 (20%) and 45 (22.5%) died in the ICU, the hospital, or before day-90, respectively. After a median follow-up of 19 months [IQR 3–45], median survival was not reached and survival at month 80 was estimated at 60% (Fig. 2). Main causes of death in the ICU were sepsis in 16 patients, cardiogenic shock in 4, cerebrovascular stroke in 2, cancers in 2, and fulminant hepatitis in 1.

By univariate analysis, predictive factors of in-hospital death were the following (Tables 1 and 2): history of peripheral arterial disease, immunosuppressive regimen without antimetabolites at admission, high gravity scores at day 1, organ failure (mechanical ventilation; vasopressive drugs; RRT; normalized prothrombin time < 50% at admission), and withdrawal of CNI in the ICU. In patients with available data, a history of positive EBV viremia or BK viruria was also predictive of death in hospital.

By multivariate analysis, SAPS2 score (OR 1.06, IC_95%_ [1.03–1.08], *p* < 0.0001) and CNI withdrawal in ICU (OR 2.65, IC_95%_ [1.11–6.32], *p* = 0.027) were the only predictive factors of death during the hospital stay. When the analysis was conducted in the sub-group of patients with available virology data (*n* = 127), the two factors that independently predicted the risk of death in hospital were the gravity SAPS2 score at admission (OR 1.07, IC_95%_ [1.03–1.11], *p* = 0.001) and a positive EBV viremia in the 6 months preceding the admission (OR 4.35, IC_95%_ [1.52–12.5], *p* = 0.006).

### Renal graft outcome

**Fig. 3:**
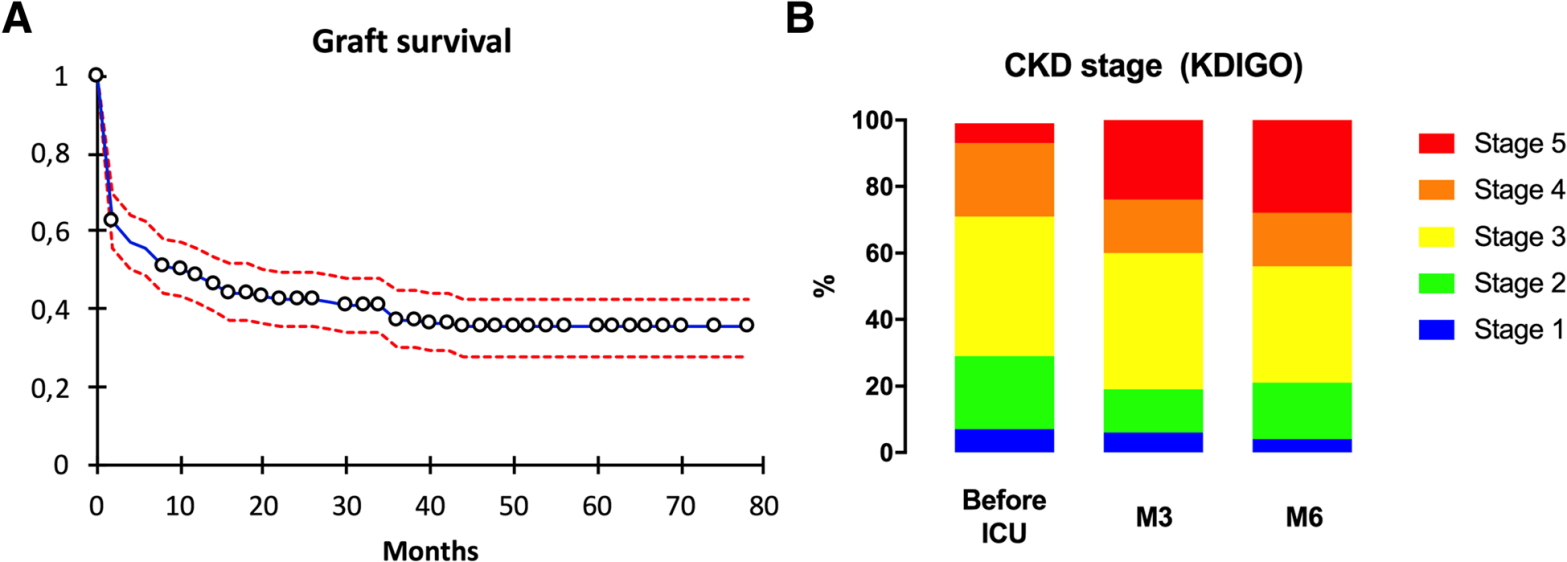
Renal outcome of the 200 kidney transplant recipients admitted to the ICU. **a**. Graft survival curve. **b**. Progression of chronic kidney disease following admission to the ICU (according to the CKD KDIGO stage)

As a secondary objective, we characterized the risk to develop AKI to CKD transition. Graft survival is shown in Fig. 3a. Median graft survival was 10 months. To better characterize the renal outcome and progression from AKI toward CKD in KTR admitted to the ICU, we studied the sub-group of patients with stable renal function at admission (i.e. admitted more than 1 month after the renal transplantation) and still alive 6 months after the admission to the ICU. In these 113 patients, median eGFR at month 6 was 35 mL/min/1.73 m^2^ [IQR 11–55], compared to 44 mL/min/1.73m^2^ [IQR 27–61] before hospitalization in the ICU (*p* = 0.002). A progression of at least one stage in the CKD KDIGO classification was observed in 34 (30.1%) and 51 (45.1%) patients at 1 and 6 months, respectively (Fig. 3b). Staging of the CKD before the admission (stage 4–5, 58% vs. stage 1–3 28.1%, *p* = 0.006) and the severity of AKI (stage 3 55.4% vs. stage 0–2 34.2%, *p* = 0.04) were significantly associated with a progression of the CKD stage at month 6.

### Immunological outcomes

Among the 119 patients with anti-HLA immunological status available before and 6 months after the admission to the ICU, 18 (15.1%) developed anti-HLA antibodies after the ICU stay, including 11 with donor-specific antibodies (DSA; 9.2%). Among these 18 patients, 6 had no detectable anti-HLA antibodies before admission, 7 had anti-HLA antibodies, and 5 had DSA. Two patients had an active ABMR, and two others stopped immunosuppressive drugs before admission. During the stay in ICU, 13 patients received RBC transfusions, and 4 needed transplant removal (urinoma, renal vein thrombosis, graft intolerance syndrome or emphysematous pyelonephritis). Two additional patients benefitted from transplant removal in the next 6 months. Patients that did not acquire HLA immunization during ICU had a significantly poorer renal prognosis (62 vs. 90% functional graft at 12 months; *p* = 0.06).

## Discussion

In this study, we reported the predictive factors of in-hospital mortality in a large cohort of 200 KTR admitted to the ICU. Risk of transition from AKI to CKD, and anti-HLA immunization, were also reported. Notwithstanding the monocentric status of this study and the inherent biases of ICU admission and management, our ICU is the referral center for KTR requiring admission to the ICU (except for neurological and cardiac surgery) in a large region in the south-west of France (~ 2.3 million inhabitants). Thus, the studied cohort accurately described the causes of KTR admission to the ICU, characteristics at presentation, and specific outcomes.

Over the study period, the estimated incidence of ICU admission (7.7%) was closed to the one reported in recent studies (4.5 to 10.2%) [2–5, 7]. Median time from renal transplantation to ICU admission was longer (41 months vs. 4.4 to 25.2 months [1–5, 7–9, 18]) except in one study [19]. The mortality rate was not influenced by the timing of ICU admission, thus confirming survival in the ICU is not associated to the characteristics of the transplantation [2, 5, 20, 21].

Our series confirms that ARF and sepsis are the main causes of ICU admission in KTR [1, 3–6, 9, 18, 20, 21]. Lung infection was the main cause of ARF with acute pulmonary edema ranking second, pointing to the high risk of sodium overload in KTR (steroids, underlying CKD or heart disease). Multiple causes of ARF were identified in 11% of patients, confirming that management of KTR should follow a dedicated workup (transplantation history, immunosuppressive regimens, underlying chronic disorders) [10, 22]. Here, 14.5% of the individuals admitted to the ICU for infection had at least two concomitant infections.

Contrasting to older studies [5, 6, 21], the mortality rate in our study (i.e., 20% in the hospital and 26.5% 6 months after the ICU stay) was lower than in unselected critically ill patients [23]. Of note, severity scores at the admission predicted mortality in hospital between 15 and 50% [24, 25]. This mortality rate, close to the one observed in two recent French studies, suggests that usual gravity scores may be undermined in KTR. Management of KTR in ICUs specialized in the field of transplantation and immunocompromised patients may also improves the outcome of these patients [3, 4]. Prognosis of solid organ transplant recipients with sepsis may be better than expected in unselected critically ill patients after adjustments on gravity scores, causes of admission, and number of organ failures [26]. Immunosuppressive drugs with reduction of the risk of hyper-inflammation state and subsequent refractory acute respiratory distress syndrome or shock may account for this discrepancy.

Interestingly, we showed for the first time that EBV replication in the months preceding the admission of KTR to the ICU was associated with a poorer outcome. EBV replication per se is not associated with a poorer outcome in unselected KTR [27]. However, chronic EBV replication may be the trigger or the consequence of T-cell exhaustion, an acquired immune paralysis observed in patients with chronic exposure to viral or cancer antigens [28]*,* associated with an increased risk of mortality or nosocomial infections in patient admitted in ICU for sepsis, burns or traumas [29]. Chronic EBV replication may thus be a surrogate marker of frailty associated with a higher risk of mortality in ICU. Further studies are warranted to confirm that EBV status at the time of admission in ICU may help to identify patients at high risk of death.

In our cohort, AKI was highly prevalent (81.5%) and 51.5% of patients required RRT, contrasting with unselected critically ill patients (19 to 57% and 4.5 to 13.5%, respectively) [30, 31]. In addition to known risk factors for the development of AKI in the ICU, the use of calcineurin inhibitors, the previous episodes of AKI and the underlying CKD, all increased the risk of AKI in KTR with acute condition [13, 14, 32, 33]. Moreover, we showed that progression of CKD after admission to the ICU is highly prevalent (30% at 1 month and 45% at 6 months in our series, compared to 12–20% at 3 months in older studies [3, 4, 11] and was well predicted by the basal CKD stage and the severity of the AKI. The high incidence of transition toward CKD in KTR, which continues beyond month 3, also points to additional and persistent renal injuries specifically encountered in this population. Whether specific management in ICU regarding the immunosuppressive regimen [10], prophylaxis of cytomegalovirus proliferation [34], and RBC transfusion policies [15] may help to overcome the risk to develop anti-HLA antibodies after admission to the ICU (15.1% of survivors in our series) in solid organ transplantation recipients need to be tested in prospective trials, because our retrospective study cannot lead to specific recommendations.

Several limitations may be underlined. First, the retrospective design of the study prevented the assessment of EBV viremia in all the KTR admitted to the ICU during the inclusion period. Nonetheless, this parameter was available in a significant number of patients (*n* = 127). Second, management of immunosuppressive regimen may vary during the ICU stay according to the patient status. Here, we discriminate patients according the maintain or the withdrawal of immunosuppressive drugs but how long patients had reduced immunosuppressive regimen was unknown in most patients. This limitation may also have modulated the risk of HLA immunization. Last, we reported here a large cohort of KTR admitted to the ICU in order to describe the different clinical scenarios that can lead to ICU admission in these patients. However, the various causes of admission introduced heterogeneity in term of both mortality and risk of immunization that need to be taken in account. It also prevents to draw firm conclusions about the optimal immunosuppressive management.

In summary, we showed that survival of KTR following admission to the ICU is predicted by the severity of the acute condition but also by viral replication (i.e., the underlying immune defense status). ICU admission is associated with a very high risk of AKI and progression toward CKD, as well as a significant risk of HLA immunization.

## Data Availability

The datasets used and/or analyzed during the current study are available from the corresponding author on reasonable request.

## Abbreviations

ABMR: Antibodies-Mediated Rejection
AKI: Acute Kidney Injury
CKD: Chronic Kidney Disease
CMV: Cytomegalovirus
CNI: Calcineurin Inhibitors
DSA: Donor-Specific Antibodies
EBV: Epstein Barr Virus
GFR: Glomerular Filtration Rate
HLA: Human Leukocytes Antigens
ICU: Intensive Care Unit
KTR: Kidney Transplant Recipients
RBC: Red Blood Cells
RRT: Renal Replacement Therapy
TCMR: T-Cells Mediated Rejection
